# Extent, pattern, and prognostic value of MGMT promotor methylation: does it differ between glioblastoma and IDH-wildtype/TERT-mutated astrocytoma?

**DOI:** 10.1007/s11060-021-03912-6

**Published:** 2021-12-13

**Authors:** Nico Teske, Philipp Karschnia, Jonathan Weller, Sebastian Siller, Mario M. Dorostkar, Jochen Herms, Louisa von Baumgarten, Joerg Christian Tonn, Niklas Thon

**Affiliations:** 1 Department of Neurosurgery, Ludwig-Maximilians-University School of Medicine, Munich, Germany; 2 German Cancer Consortium (DKTK), Partner Site Munich, Munich, Germany; 3 Center for Neuropathology and Prion Research, Ludwig-Maximilians-University School of Medicine, Munich, Germany; 4 Department of Neurology, Ludwig-Maximilians-University School of Medicine, Munich, Germany; 5 Department of Neurosurgery, Division of Neuro-Oncology, Ludwig-Maximilians-University School of Medicine, Marchioninistrasse 15, 81377 Munich, Germany

**Keywords:** WHO CNS 2021, cIMPACT, TERT, IDH wildtype, Glioma

## Abstract

**Introduction:**

The cIMPACT-NOW update 6 first introduced glioblastoma diagnosis based on the combination of IDH-wildtype (*IDHwt*) status and TERT promotor mutation (*pTERTmut*). In glioblastoma as defined by histopathology according to the WHO 2016 classification, MGMT promotor status is associated with outcome. Whether this is also true in glioblastoma defined by molecular markers is yet unclear.

**Methods:**

We searched the institutional database for patients with: (1) glioblastoma defined by histopathology; and (2) *IDHwt* astrocytoma with *pTERTmut*. MGMT promotor methylation was analysed using methylation-specific PCR and Sanger sequencing of CpG sites within the MGMT promotor region.

**Results:**

We identified 224 patients with glioblastoma diagnosed based on histopathology, and 54 patients with *IDHwt* astrocytoma with *pTERTmut* (19 astrocytomas WHO grade II and 38 astrocytomas WHO grade III). There was no difference in the number of MGMT methylated tumors between the two cohorts as determined per PCR, and also neither the number nor the pattern of methylated CpG sites differed as determined per Sanger sequencing. Progression-free (PFS) and overall survival (OS) was similar between the two cohorts when treated with radio- or chemotherapy. In both cohorts, higher numbers of methylated CpG sites were associated with favourable outcome.

**Conclusions:**

Extent and pattern of methylated CpG sites are similar in glioblastoma and *IDHwt* astrocytoma with *pTERTmut*. In both tumor entities, higher numbers of methylated CpG sites appear associated with more favourable outcome. Evaluation in larger prospective cohorts is warranted.

## Introduction

In 2016, the World Health Organization (WHO) revised the classification of central nervous system (CNS) tumors which, for the first time, incorporated both histological characteristics as well as molecular features [1]. Since the introduction of the WHO 2016 classification, ongoing advances have led to an increasing understanding of brain tumor molecular pathogenesis and its clinical impact on patients’ outcome. The Consortium to Inform Molecular and Practical Approaches to CNS Tumor Taxonomy—(cIMPACT‐NOW) was founded to review and integrate those advances into clinical practice between WHO updates. The cIMPACT-NOW update 6 has proposed to reclassify isocitrate dehydrogenase 1/2 wildtype (*IDHwt*) diffuse astrocytomas as glioblastoma if they present with either (1) telomerase reverse transcriptase (TERT) promotor mutation (*pTERTmut*); (2) epidermal growth factor receptor (EGFR) gene amplification; or (3) whole chromosome 7 gain and whole chromosome 10 loss (+ 7/− 10) [2]. Such tumors were found to have clinical outcomes similar to those of glioblastoma as defined per histopathology, and as expected, the recently published WHO 2021 classification has incorporated the diagnosis of glioblastoma based on molecular markers [3, 4].

Methylation of the promotor region of the O6-methylguanine-DNA-methlytransferase (MGMT) gene is another molecular marker associated with favourable prognosis and response to alkylating chemotherapy in glioblastoma [5, 6]. In the prospective CATNON trial (which compares radiotherapy with or without chemotherapy), a subgroup analysis of *IDHwt* astrocytoma with molecular features of glioblastoma demonstrated improved survival for tumors with MGMT promotor methylation (but surprisingly did not find evidence for beneficial effects of alkylating chemotherapy among methylated tumors) [7]. We recently reported on a large cohort of gliomas WHO grade II, and found that a higher number of methylated CpG sites within the MGMT promotor region also represents a positive prognostic factor for outcome in gliomas of lower grades [8]. Of note, a subgroup analysis of our cohort showed that the prognostic value of MGMT promotor methylation was only retained in *IDHwt* astrocytomas, but not in IDH-mutant glioma with or without 1p19q co-deletion. However, the subgroup of *IDHwt* astrocytomas included in our previous study was limited given its small sample size of only 20 patients and missing TERT status.

In the present study, we describe a molecularly well-defined cohort of 57 *IDHwt* astrocytoma with *pTERTmut* who were consecutively treated at a single academic neuro-oncology centre. Based upon the comparison with a group of 224 glioblastoma patients defined per histopathology according to the WHO 2016 classification, we aim to describe pattern and extend of MGMT promotor methylation in *IDHwt* astrocytoma with *pTERTmut* and its association with survival in the presence of chemo- and radiotherapy.

## Materials and methods

### Study population

Study design and methods were approved by the Institutional Review Board of the Ludwig Maximilians University in Munich, Germany, and patient consent was waived (AZ 20-650). We retrospectively searched the institutional database of the Center for Neuro-Oncology at the Ludwig Maximilians University School of Medicine for adult patients seen between 2004 and 2014 with: (1) *IDHwt* glioblastoma WHO grade IV as defined by histopathology according to the WHO 2016 classification [1]; and (2) *IDHwt* astrocytoma with *pTERTmut* in the absence of classical histological hallmarks (corresponding to WHO grade II and III *IDHwt* according to the WHO 2016 classification, but to WHO grade 4 according to the cIMPACT-NOW update 6 and WHO 2021 classification) [2, 4]. Histopathologic diagnosis was based upon tissue sampled during microsurgical tumor removal, or stereotactic biopsy in lesions where safe resection appeared not feasible. Patients with IDH1/2 mutations or in which IDH status was unavailable for review were excluded from the study. Diagnostic and treatment decisions were based upon interdisciplinary brain tumor board recommendations and patient preference. Follow-up imaging and surveillance scans were obtained per institutional guidelines with follow-up imaging every three to six months or in case of any clinical detoriation [9]. We collected demographic and clinical information, histopathology, molecular markers and other diagnostic findings, treatment specifics and clinical outcome. Complete resection of contrast-enhancing tumor was defined as previously proposed in the classification by Karschnia et al. [10] Database closure in this study was December 1, 2020.

### MGMT promotor methylation and molecular markers

MGMT promotor status was analysed using the following two methods: (1) methylation-specific polymerase chain reaction (MSP) and (2) Sanger sequencing of the Cytosine-Guanine dinucleotide (CpG) sites 74–98 within the MGMT promotor region as previously described [11, 12]. CpG site methylation was defined as ratio of cytosine/thymine peak > 50%. The total number of methylated CpG sites was calculated for each patient.

IDH 1/2 mutation status was assessed per pyrosequencing, and TERT promotor mutation status was retrospectively analysed using Sanger sequencing as previously described for the purpose of the present study [13, 14]. TERT promotor mutation status was not routinely tested in histopathological GBMs.

### Statistical analysis

Categorical variables are described in absolute numbers and percent points. Relationships between two or more categorical variables were assessed using the chi-square test. For numerical data, the D' Agostino-Pearson omnibus normality test was used to test for normal distribution. In case of parametric data, differences between two groups were analysed by the unpaired Student’s t test. Mann–Whitney U-test was used to assess differences between two groups in case of non-parametric data. Differences among more than two groups were analysed by ANOVA. If not indicated otherwise, all values are expressed as mean ± standard error of the mean and range is given. For survival analyses, patients were followed until day of database closure (December 1, 2020) or death. Patients lost to follow-up were censored at day of last follow-up. Date of diagnosis was set as date of pathological diagnosis. Date of radiographic progression was defined as date when diagnosis of radiographic progression according to RANO criteria was made, or tumor-related death. Overall survival was defined as interval from diagnosis to tumor-related death. Kaplan–Meier survival analysis and log-rank test were used to calculate follow-up, survival, and predictors of outcome. Statistical analyses were performed using Prism statistical software (Prism 9.0; GraphPad Software Inc., San Diego, CA, USA). The significance level was set at *p* ≤ 0.05.

## Results

### Study population

A total of 281 patients were identified and included in the present study. We encountered 224 glioblastomas WHO 2016 grade IV (80%, all *IDHwt*; hereafter referred to as ‚histopathological GBM′) and 57 *IDHwt* astrocytomas with *pTERTmut* (20%; hereafter referred to as ‚molecular GBM′) (Table 1). The latter cohort consisted of 18 diffuse astrocytomas *IDHwt* WHO 2016 grade II (18/57 patients, 32%), 38 anaplastic astrocytomas *IDHwt* WHO 2016 grade III (38/57 patients; 67%), and 1 gemistocytic astrocytoma *IDHwt* WHO 2016 grade II (1/57 patients; 2%).

**Table 1:** Patient characteristics for glioblastoma and *IDHwt* astrocytoma with *pTERTmut*

	Glioblastoma WHO°IV	*IDHwt* astrocytomas with *pTERTmut*	Total	*p-*value
**Overall, n (%)**	224 (80%)	57 (20%)	281	
**Age, years**				
< 18	1 (0%)	0	1 (0%)	0.695
18–35	11 (5%)	0	11 (4%)	
36–50	34 (15%)	16 (28%)	50 (18%)	
51–65	101 (45%)	22 (39%)	123 (44%)	
> 65	77 (34%)	19 (33%)	96 (34%)	
**Gender**				
Female	80 (36%)	23 (40%)	103 (37%)	0.518
Male	144 (64%)	34 (60%)	178 (63%)	
**KPS, %**				
< 90	127 (57%)	23 (40%)	150 (53%)	*0.027
90–100	97 (43%)	34 (60%)	131 (47%)	
**Histopathology**				
Diffuse AST WHO°II	0	18 (32%)	18 (6%)	
Anaplastic AST WHO°III	0	38 (67%)	38 (14%)	
Gemistocytic AST WHO°II	0	1 (2%)	1 (0%)	
GBM WHO°IV	224 (100%)	0	224 (80%)	
**Methylated CpG sites**				
0–8	99 (44%)	21 (37%)	120 (43%)	0.527
9–16	40 (18%)	13 (23%)	53 (19%)	
17–25	85 (38%)	23 (40%)	108 (38%)	
**First-line therapy**				
Chemotherapy				*0.001
TMZ	7 (3%)	16 (28%)	23 (8%)	
PC	0	2 (4%)	2 (1%)	
Radiotherapy	0	10 (18%)	10 (4%)	*0.001
Radiochemotherapy	216 (96%)	15 (26%)	231 (82%)	*0.001
Brachytherapy	1 (0%)	4 (7%)	6 (2%)	*0.001
Wait-and-scan	0	10 (18%)	10 (4%)	*0.001

Characteristics are given for patients with glioblastoma, IDH-wildtype, WHO grade IV (n = 224) and *IDHwt* astrocytoma with *pTERTmut,* WHO grade II and III (n = 57); and are summarized for all patients (total; n = 281)

*CpG*: cytosine-guanine dinucleotide, *IDHwt*: isocitrate dehydrogenase 1/2 wildtype, *KPS*: Karnofsky performance score, *MGMT*: O6-methylguanine-DNA methyltransferase promotor, *PC*: procarbazine, lomustine, *TMZ:* temozolomide, *TERT*: telomerase reverse transcriptase promotor, *pTERTmut:* TERT promotor mutation, *TMZ*: temozolomide

Asterisks indicate *p ≤ 0.05

### Demographic and clinical findings

Among the two cohorts, median patient age at diagnosis was similar (histopathological GBM: 59 ± 0.8 years, range 13–86 years and molecular GBM: 59 ± 1.4 years, range 39–81; *p* = 0.695). Male-to-female ratio was 1:0.6 in histopathological GBM and 1:0.7 in molecular GBM (*p* = 0.518). Karnofsky performance score was significantly higher in patients with molecular GBM (90%; range 60–90% vs. 80%; 40–100 in histopathological GBM) (*p = 0.027).

### MGMT promotor methylation

**Fig. 1:**
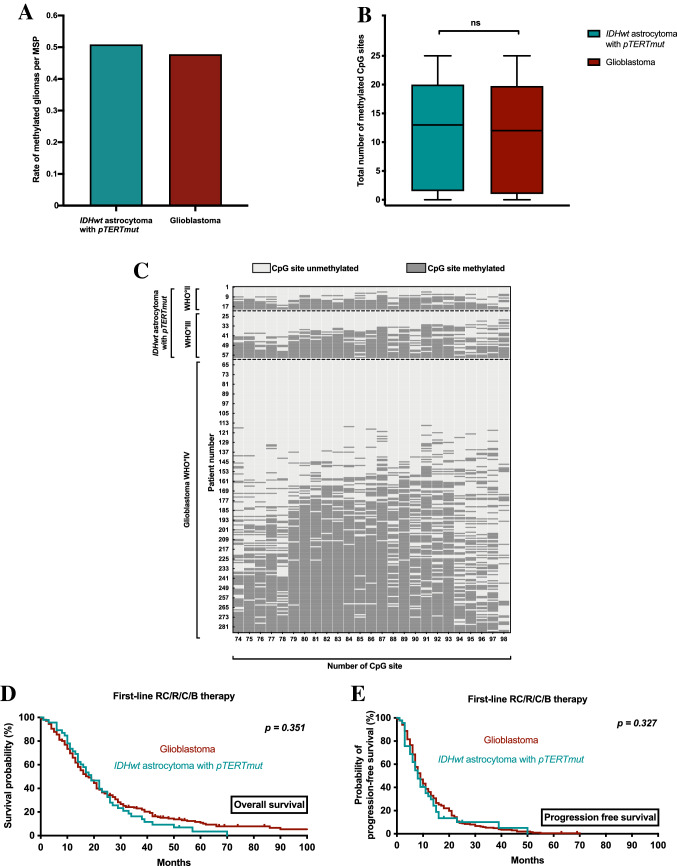
Survival and extent of MGMT promotor methylation in glioblastoma and *IDHwt* astrocytoma with *pTERTmut*. **A** Rate of methylated tumors per MSP in patients with *IDHwt* astrocytoma with *pTERTmut*, WHO grade II and III (cyan) and glioblastoma (red). **B** Number of methylated CpG sites in patients with *IDHwt* astrocytoma with *pTERTmut*, WHO grade II and III (cyan) and gliobastoma (red). Median, interquartile range, and total range are given. **C** Methylation pattern of CpG sites 74–98 within the MGMT promotor region in patients with *IDHwt* astrocytoma with *pTERTmut*, WHO grade II (n = 19) and WHO grade III (n = 38), and glioblastoma WHO grade IV (n = 224). Each row corresponds to an individual patient, and each column to a different CpG site. Dark grey rectangles represent methylated sites and light grey rectangles represent unmethylated sites. **D/E** Kaplan–Meier estimates of overall survival (**D**) and radiographic progression-free survival (**E**) in the entire cohort treated with any medical therapy. Patients were stratified into *IDHwt* astrocytoma with *pTERTmut*, WHO grade II and III (cyan) and glioblastoma, WHO grade IV (red). *B therapy:* brachytherapy; *C therapy:* chemotherapy; *CpG:* Cytosine-Guanine dinucleotide; *IDHwt:* isocitrate dehydrogenase 1/2 wildtype; *pTERTmut*: TERT promotor mutation; *R therapy:* radiotherapy; *RC therapy:* radiochemotherapy

MSP and Sanger sequencing data was available for review for all patients. Binary analysis of MGMT promotor methylation with MSP showed comparable methylation rates with 48.4% methylation in the entire cohort, 47.8% in histopathological GBM, and 50.9% in molecular GBM (*p* = 0.675) (Fig. 1A). In the entire cohort, the mean number of methylated CpG sites was 11.3 ± 0.5 (45 ± 2.1% of 25 CpG sites) and did strongly vary between individual patients (range 0–25). The mean number of methylated CpG sites in histopathological GBM was 11.9 ± 1.2 (47.6 ± 4.7%; range 0–25) and did not significantly differ when compared to 11.1 ± 0.6 (44.3 ± 2.7%; range 0–25) in molecular GBM (*p* = 0.545) (Fig. 1B). Also, the range in the individual number of methylated CpG sites was identical in both groups (0–25 CpG sites). Moreover, mean number of methylated CpG sites was similar when allocating gliomas according to WHO 2016 classification with 11.4 ± 2.1 (45.7 ± 8.5% of 25 CpG sites) in WHO grade II, 12.2 ± 1.4 (48.6 ± 5.7% of 25 CpG sites) in WHO grade III, and 11.1 ± 0.6 (44.3 ± 2.7%; range 0–25) in WHO grade IV (*p* = 0.784).

Of note, histopathological and molecular GBM showed a similar methylation pattern with some CpG sites such as number 87 and 91 being more frequently found to be methylated than others (Fig. 1C).

### Treatment and outcome

Diagnosis was made by microsurgical tumor resection or stereotactic biopsy. Following tissue-based diagnosis, first-line therapeutic management of all gliomas included chemotherapy (temozolomide or procarbazine/lomustine), involved-field radiotherapy, radiochemotherapy, interstitial brachytherapy, and wait-and-scan approaches (Table 1). Radiochemotherapy with temozolomide was most often provided in histopathological GBM (216/224 patients, 96%), followed by alkylating chemotherapy with temozolomide in the absence of radiotherapy (7/224 patients, 3%). Interstitial brachytherapy was provided in 1 patient (< 1%). Molecular GBM received a more diverse first-line therapy, most often consisting of alkylating chemotherapy (overall: 18/57 patients, 32%; temozolomide: 16/57 patients, 28%; procarbazine/lomustine: 2/57 patients, 4%) or radiochemotherapy (15/57 patients, 26%). Of note, 10/57 (18%) patients with a molecular GBM received wait-and-scan approaches after initial biopsy, the majority being assigned as WHO grade II tumors (8/57 patients, 14%). Microsurgical tumor resection was more frequently provided in histopathological GBM (94/224 patients, 42%) than in molecular GBM (3/57 patients, 5%). In resected tumors, gross total tumor resection was most often achieved (histopathological GBM: 53/94 patients, 56%; molecular GBM: 2/3 patients, 66%) All other patients received stereotactic biopsy for diagnostic purposes. There was no clear difference in regard of therapy provided after tumor progression between patients in both cohorts.

Median follow-up was 24 months (range 0–142 months). In the entire group, median time to radiographic progression was 9 months (range 0–71 months) and median overall survival was 19 months (range 1–142 months). Next, we aimed to compare overall survival and radiographic progression free survival in patients with first-line medical therapy including radio- or chemotherapy of any kind (radiochemotherapy, alkylating chemotherapy, radiotherapy, interstitial brachytherapy). Median overall survival was identical in both cohorts with 19 months (*p* = 0.356); median time to radiographic progression was similar in both cohorts with 9 months in histopathological GBM vs. 8 months in molecular GBM (n = 224 vs. 47; *p* = 0.327) (Fig. 1D, E).

In patients treated with any chemotherapy (including radiochemotherapy) at first line, molecular GBM showed a similar median overall survival and progression free survival in comparison to histopathological GBM (molecular GBM n = 33, histopathological GBM n = 223; OS 22 vs. 19 months, *p* = 0.625; PFS 8 vs. 9 months, *p* = 0.179).

### Association of MGMT promotor methylation with outcome

**Fig. 2:**
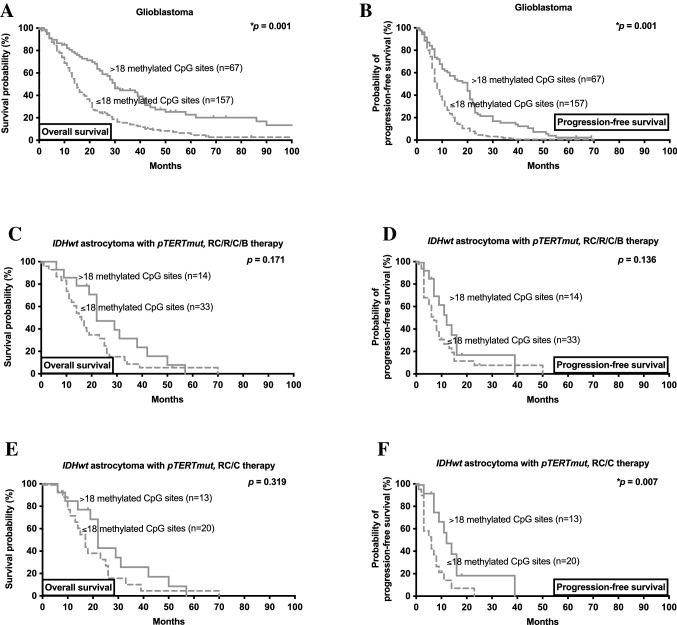
MGMT as a marker for survival and disease progression in glioblastoma and *IDHwt* astrocytoma with *pTERTmut*. **A/C** Kaplan–Meier estimates of overall survival in glioblastoma and *IDHwt* astrocytoma with *pTERTmut* treated with any form of radio-/chemotherapy. Curves are displayed for patients with > 18 methylated CpG sites (straight lines) and ≤ 18 methylated CpG sites (dottes lines) **B/D** Kaplan–Meier estimates of radiographic progression-free survival in glioblastoma and *IDHwt* astrocytoma with *pTERTmut* treated with any form of radio-/chemotherapy. Curves are displayed for patients with > 18 methylated CpG sites (straight lines) and ≤ 18 methylated CpG sites (dottes lines). **E/F** Kaplan–Meier estimates of overall survival (**E**) and radiographic progression-free survival (**F**) in *IDHwt* astrocytoma with *pTERTmut* treated with first-line radiochemotherapy or chemotherapy. Curves are displayed for patients with > 18 methylated CpG sites (straight lines) and ≤ 18 methylated CpG sites (dottes lines). Tick marks indicate censored patients. *B therapy:* brachytherapy; *C therapy:* chemotherapy; *CpG:* Cytosine-Guanine dinucleotide; *IDHwt:* isocitrate dehydrogenase 1/2 wildtype; *pTERTmut*: TERT promotor mutation; *R therapy:* radiotherapy; *RC therapy:* radiochemotherapy

To analyse whether MGMT promotor methylation was associated with outcome in the presence of radio- or chemotherapy, patients who received medical treatment including radiochemotherapy, radiotherapy, chemotherapy and brachytherapy were stratified according to number of methylated CpG sites. A larger number of methylated CpG sites was associated with favourable outcome in glioblastoma patients (Table 2): a total of > 18 methylated CpG sites was a significant cutoff for improved overall survival (15 vs. 30 months, hazard ratio 0.49, *p* = *0.001) and the most significant cutoff for longer time to radiographic progression (8 vs. 20 months, hazard ratio 0.48, *p* = *0.001) (Fig. 2A, B).

**Table 2:** Number of methylated CpG sites within the MGMT promotor region as a prognostic factor in glioblastoma

Number of methylated CpG sites (*patients at risk*)	Radiographic progression-free survival			Overall survival		
n = 224	Hazard ratio	95% confidence interval of HR	*p*-value	Hazard ratio	95% confidence interval of HR	*p*-value
0 (44) vs. ≥ 1 (180)	0.60	0.4–0.9	*0.001	0.55	0.4–0.8	*0.001
≤ 1 (62) vs. ≥ 2 (162)	0.59	0.4–0.8	*0.001	0.52	0.4–0.7	*0.001
≤ 2 (71) vs. ≥ 3 (153)	0.53	0.4–0.7	*0.001	0.45	0.3–0.6	*0.001
≤ 3 (75) vs. ≥ 4 (149)	0.56	0.4–0.8	*0.001	0.47	0.3–0.7	*0.001
≤ 4 (80) vs. ≥ 5 (144)	0.53	0.4–0.7	*0.001	0.44	0.3–0.6	*0.001
≤ 5 (86) vs. ≥ 6 (138)	0.53	0.4–0.7	*0.001	0.45	0.3–0.6	*0.001
≤ 6 (91) vs. ≥ 7 (133)	0.53	0.4–0.7	*0.001	0.45	0.3–0.6	*0.001
≤ 7 (92) vs. ≥ 8 (132)	0.53	0.4–0.7	*0.001	0.45	0.3–0.6	*0.001
≤ 8 (99) vs. ≥ 9 (125)	0.51	0.4–0.7	*0.001	0.44	0.3–0.6	*0.001
≤ 10 (103) vs. ≥ 11 (121)	0.49	0.4–0.6	*0.001	0.43	0.3–0.6	*0.001
≤ 11 (107) vs. ≥ 12 (117)	0.48	0.4–0.6	*0.001	0.43	0.3–0.6	*0.001
≤ 12 (116) vs. ≥ 13 (108)	0.47	0.4–0.6	*0.001	0.45	0.3–0.6	*0.001
≤ 13 (118) vs. ≥ 14 (106)	0.47	0.4–0.6	*0.001	0.46	0.3–0.6	*0.001
≤ 14 (123) vs. ≥ 15 (101)	0.46	0.4–0.6	*0.001	0.49	0.4–0.6	*0.001
≤ 15 (129) vs. ≥ 16 (95)	0.46	0.4–0.6	*0.001	0.48	0.4–0.6	*0.001
≤ 16 (139) vs. ≥ 17 (85)	0.45	0.3–0.6	*0.001	0.50	0.4–0.7	*0.001
≤ 17 (147) vs. ≥ 18 (77)	0.45	0.3–0.6	*0.001	0.47	0.4–0.6	*0.001
≤ 18 (157) vs. ≥ 19 (67)	0.48	0.4–0.6	*0.001	0.49	0.4–0.7	*0.001
≤ 19 (168) vs. ≥ 20 (56)	0.53	0.4–0.7	*0.001	0.52	0.4–0.7	*0.001
≤ 20 (180) vs. ≥ 21 (44)	0.50	0.3–0.7	*0.001	0.48	0.4–0.7	*0.001
≤ 21 (193) vs. ≥ 22 (31)	0.57	0.4–0.8	*0.002	0.60	0.4–0.9	*0.013
≤ 22 (204) vs. ≥ 23 (20)	0.50	0.3–0.7	*0.001	0.53	0.3–0.8	*0.012
≤ 23 (215) vs. ≥ 24 (9)	0.55	0.3–0.9	0.057	0.57	0.3–1.0	0.123
≤ 24 (220) vs. ≥ 25 (4)	0.46	0.2–0.9	0.092	0.69	0.3–1.6	0.447

Univariate analysis for radiographic progression-free and overall survival was performed among patients with glioblastoma, IHD-wildtype, WHO grade IV (n = 224). Number of methylated CpG sites was tested as dichotomous variable. Number of patients at risk is indicated. Hazard ratio, 95% confidence interval of hazard ratio, and *p*-value are given

*CpG:* cytosine-guanine dinucleotide, *HR*: hazard ratio

Asterisks indicate *p ≤ 0.05

In patients with molecular GBM treated with radio-/chemotherapy, no significant correlation between number of methylated CpG sites and overall survival or time to radiographic progression was seen, respectively (Table 3); but a relevant trend towards better outcome was seen in tumors with a total of > 18 methylated CpG sites (HR 0.65 for overall survival, *p* = 0.171; Fig. 2C, D). To minimize bias from extent of resection we also compared patients with molecular GBM treated with radio-/chemotherapy who only received stereotactic biopsy (and no surgical tumor resection). Here, no significant difference in overall survival (22 vs. 16 months, HR 0.67, *p* = 0.216) nor time to radiographic progression (11 vs. 6 months, HR 0.67, *p* = 0.238) was seen for patients with more or less than 18 methylated CpG sites.

**Table 3:** Number of methylated CpG sites within the MGMT promotor region in *IDHwt* astrocytoma with *pTERTmut*

Number of methylated CpG sites (*patients at risk*)	Radiographic progression-free survival			Overall survival		
n = 47	Hazard ratio	95% confidence interval of HR	*p*-value	Hazard ratio	95% confidence interval of HR	*p*-value
0 (8) vs. ≥ 1 (39)	1.46	0.7–3.0	0.311	0.94	0.4–2.1	0.871
≤ 1 (12) vs. ≥ 2 (35)	1.38	0.7–2.7	0.334	0.88	0.4–1.8	0.699
≤ 2 (14) vs. ≥ 3 (33)	1.17	0.6–2.3	0.615	0.82	0.4–1.7	0.557
≤ 4 (15) vs. ≥ 5 (32)	1.12	0.6–2.3	0.610	0.74	0.4–1.5	0.344
≤ 5 (17) vs. ≥ 6 (30)	1.08	0.6–2.1	0.808	0.73	0.4–1.4	0.304
≤ 7 (18) vs. ≥ 8 (29)	1.06	0.6–2.0	0.856	0.72	0.4–1.4	0.271
≤ 9 (19) vs. ≥ 10 (28)	0.97	0.5–1.9	0.931	0.66	0.3–1.3	0.163
≤ 10 (21) vs. ≥ 11 (26)	0.83	0.4–1.6	0.537	0.92	0.5–1.7	0.783
≤ 11 (23) vs. ≥ 12 (24)	0.89	0.5–1.7	0.681	0.95	0.5–1.7	0.864
≤ 13 (24) vs. ≥ 14 (23)	0.86	0.5–1.6	0.606	0.90	0.5–1.6	0.732
≤ 14 (25) vs. ≥ 15 (22)	0.83	0.4–1.6	0.535	0.91	0.5–1.7	0.751
≤ 15 (27) vs. ≥ 16 (20)	0.76	0.4–1.4	0.351	0.80	0.4–1.5	0.448
≤ 17 (30) vs. ≥ 18 (17)	0.62	0.3–1.2	0.125	0.69	0.4–1.3	0.218
≤ 18 (33) vs. ≥ 19 (14)	0.61	0.3–1.2	0.136	0.65	0.4–1.2	0.171
≤ 19 (34) vs. ≥ 20 (13)	0.72	0.4–1.4	0.326	0.78	0.4–1.5	0.450
≤ 20 (37) vs. ≥ 21 (10)	0.75	0.4–1.6	0.459	1.05	0.5–2.2	0.888
≤ 21 (39) vs. ≥ 22 (9)	0.58	0.3–1.3	0.216	1.13	0.5–2.6	0.759
≤ 22 (42) vs. ≥ 23 (5)	0.31	0.1–0.8	0.068	1.34	0.4–4.3	0.561
≤ 23 (43) vs. ≥ 24 (4)	0.34	0.1–0.9	0.095	1.02	0.3–3.3	0.970
≤ 24 (45) vs. ≥ 25 (2)	n.a	n.a	0.071	1.24	0.1–11.2	0.828

Univariate analysis for radiographic progression-free and overall survival was performed among patients with *IDHwt* astrocytoma with *pTERTmut* treated with radio- or chemotherapy of any kind (n = 47). Number of methylated CpG sites was tested as dichotomous variable. Number of patients at risk is indicated. Hazard ratio, 95% confidence interval of hazard ratio, and *p*-value are given

*CpG:* cytosine-guanine dinucleotide, *HR*: hazard ratio, *n.a.:* not applicable, *pTERTmut*: TERT promotor mutation

Next, we aimed to analyse whether MGMT promotor methylation was associated with outcome in patients receiving alkylating chemotherapy (including radiochemotherapy) in molecular GBM. We divided the cohort into individuals with msore (n = 13) or less (n = 20) than 18 methylated CpG sites (in analogy to the optimal cut-off, see above; Fig. 2E, F). Of interest, higher number of methylated CpG sites were associated with longer time to radiographic progression (12 vs. 6 months, *p = 0.007). Overall survival was longer in patients with > 18 methylated CpG sites compared with ≤ 18 methylated CpG sites, but without statistical significance (22 vs. 17 months, *p* = 0.319). Similar findings were made when only comparing patients who received stereotactic biopsy without microsurgical tumor resection (OS 22 vs. 17 months, *p* = 0.366; PFS 12 vs. 6 months, *p* = *0.034).

In addition, all patients with molecular GBM with re-exposure to alkylating chemotherapy (including radiochemotherapy) for post-progression therapy (n = 28) were stratified according to number of methylated CpG sites as above. Here, no significant difference in outcome between patients with > 18 methylated CpG sites (n = 9) and patients with ≤ 18 methylated CpG sites (n = 19) was seen (15 vs. 13 months, *p* = 0.917).

## Discussion

cIMPACT-NOW first introduced diagnosis of glioblastoma, WHO grade 4 based upon the presence of molecular features in *IDHwt* astrocytoma WHO grade II and III (according to WHO 2016) even in the absence of classical histological hallmarks. Also, the recently published WHO 2021 classification now incorporates grading of gliomas based on such molecular markers [4]. Methylation of the MGMT promotor region is an essential molecular marker for outcome and response to alkylating chemotherapy in histologically defined glioblastoma [5, 15]. However, its role in *IDHwt* astrocytoma with *pTERTmut* is less well established. A subgroup analysis of the CATNON trial with 154 *IDHwt* astrocytoma with molecular features of glioblastoma showed that MGMT promotor methylation was prognostic for overall survival but not predictive for temozolomide chemotherapy in this cohort [7]. Data from the NOA-04 trial demonstrated the positive predictive value of MGMT promotor methylation in *IDHwt* astrocytomas WHO grade III but did not test for molecular features of glioblastoma like TERT promotor mutation [6]. Of interest, several studies found a prognostic interaction of TERT promoter mutation with MGMT promoter methylation in patients with *IDHwt* glioblastoma treated with radiochemotherapy [16, 17]. Furthermore, a recent meta-analysis with low-grade glioma as well as glioblastoma patients including the aforementioned studies showed that among TERT promotor mutated low-grade gliomas, MGMT promotor methylation was associated with improved overall survival [18]. However, no IDH-status was evaluated in this retrospective subgroup analysis. Here, we focused on detailing our institutional experience on the role of MGMT promotor methylation in *IDHwt* astrocytomas with *pTERTmut*.

We found that extent of MGMT promotor methylation was similar in both histologically defined glioblastoma as well as *IDHwt* astrocytomas with *pTERTmut*. Methylation rates were comparable to those for glioblastoma reported in the literature [19]. A larger number of methylated CpG sites was prognostic for improved overall survival as well as longer time to radiographic progression in glioblastoma. In molecular GBM treated with alkylating chemotherapy including radiochemotherapy*,* MGMT promotor methylation was associated with improved progression free survival (statistical significance for overall survival was not reached in patients with molecular GBM, potentially due to the relatively small sample size). Interestingly, MGMT methylation of CpG sites 74–98 using Sanger sequencing showed a similar pattern across histopathological and molecular GBM patients with certain CpG sites such as 87 being more frequently found to be methylated, thus underlining their molecular similarities. In a retrospective study of histopathological GBM patients treated with radiochemotherapy, Sanger sequencing analysis showed a potential linear correlation of methylated CpG sites with outcome highlighting its additional value in contrast to conventional methods [20]. Of note, Sanger sequencing and other methods assessing MGMT promotor methylation status correlate with low MGMT mRNA expression levels but not necessarily MGMT protein levels, indicating post-transcriptional regulation of MGMT that can affect predictive and prognostic value of MGMT promotor methylation status [21]. Overall, extent of MGMT promotor methylation was comparable between histopathological and molecular GBM treated with radiotherapy or radiochemotherapy. However, particularly its predictive role for response to alkylating chemotherapy seems less clear and warrants evaluation in large prospective trials.

Furthermore, we aimed to define a MGMT promotor methylation cut-off point for strongest prognostic value in molecular GBM as well as histopathological GBM patients using Sanger sequencing of 25 CpG sites. Interestingly, the calculated MGMT promotor methylation cut-off point predicting longer progression-free survival was higher (≥ 18/25 CpG sites, ≥ 72%) in comparison to the cut-off point predicting improved overall survival (≥ 11/25 CpG sites, ≥ 44%) in histopathological GBM patients. A prognostic cut-off point in molecular GBM patients could not be calculated but a as a trend, a non-significant better outcome was also seen in tumors with a total of > 18 methylated CpG sites (≥ 76%), regardless of extent of resection. Other cut-off points which have been validated in studies by others range from a mean MGMT promotor methylation of 7% to 30% [22, 23] which is lower than our calculated cut-off points. Of interest, mean number of methylated CpG sites in glioblastoma as well as *IDHwt* astrocytoma with *pTERTmut* were comparable to mean number of methylated CpG sites in a cohort of glioma WHO grade II recently described by our group [8], suggesting extent of MGMT promotor methylation to be independent of histopathological WHO grade and may rather depend on molecular markers. Technical cut-off values for the distinction of methylated versus unmethylated cases usually would be set at the nadir of the distribution. However, given the heterogenous methylation patterns in gliomas, there appears to be prognostic and predictive uncertainty for patients with an intermediate number of methylated CpG sites. Finding consensus on reliable cut-off values remains to be found and will need prospective validation in the future. [24].

As it is the case in our neuro-oncology center for all glioma patients, Wick et al. proposed to test for MGMT promotor methylation using two distinct methods for a better discrimination in patients with a “grey zone” methylation status [19]. Consequently, results from both methods can then be added to guide therapeutic management. In our cohort, MGMT promotor methylation status was analysed by two commonly used methods comprising of 1) MSP and 2) Sanger sequencing. Both showed comparable methylation rates in our entire cohort, as well as subgroup analysis. Of note, both methods described in this study are semi-quantitative analysis methods to assess MGMT promotor methylation and thus underlie a more subjective interpretation rather than fully quantitative methods such as pyrosequencing. A recent comprehensive meta-analysis examining studies using different methods for MGMT promotor methylation testing in glioblastoma patients treated with temozolomide showed MSP and pyrosequencing to be superior to immunohistochemistry for MGMT protein but did not provide evidence for best CpG site threshold [25]. A gold standard to distinguish between patients with and without MGMT promotor methylation remains to be defined in *IDHwt* astrocytomas with *pTERTmut*, but a combination of different methods seems to be a feasible approach.

Next, we reviewed treatment-algorithms in our cohort. Management was distinctly different in both subgroups. Whereas histopathological GBM patients most commonly received microsurgical tumor resection followed by radiochemotherapy with temozolomide, treatment of molecular GBM patients was more diverse. An unusually high amount of 18% received a wait-and-scan approach until first recurrence. In part, patient’s preference and thus shared decision making in treatment management has to be accounted for. More importantly, however, most patients with a wait-and-scan approach in our cohort were diagnosed during a time when significance of molecular markers like IDH mutation and TERT promotor mutation were less well established and treatment strategies in low grade gliomas varied from wait-and-scan to complete resection of all visible tumor on MRI. Consequently, tumor tissue from initial biopsy or surgery was most often tested for TERT promotor mutation at time of recurrence or even retrospectively for the purpose of the present study (9/10 patients with wait-and-scan approach, 90%). In addition, most gliomas in this subgroup were classified as WHO grade II thus explaining treatment decisions in these patients. IDH 1/2 mutation status was assessed per pyrosequencing, and TERT promotor mutation status was retrospectively analysed using Sanger sequencing as previously described for the purpose of the present study. As significance of molecular markers in our daily treatment decisions increase and suitable diagnostics are more frequently incorporated in the routine pathological tumor workup, it will become increasingly important to address treatment differences and compare similar molecular tumor signatures in future studies to minimize bias.

Furthermore, stereotactic biopsy was the preferred choice for tumor diagnosis in molecular GBMs whereas histopathological GBM patients often received microsurgical tumor resection. To what extent differences in extent of resection (biopsy vs. gross total resection vs. subtotal resection) may play a role in patients’ outcome as well as its association with MGMT promotor methylation remains uncertain. Also, different terminology to describe extent of resection across clinical trials have made it difficult to perform comparative analysis between study centers. [10].

Overall survival as well as radiographic progression-free survival was similar in both cohorts and consistent with previously reported data [26, 27]. On a cautionary note, progression-free survival was similar in both subgroups although glioblastoma patients more often received aggressive therapy with tumor resection and radiochemotherapy. Patients with *IDHwt* astrocytoma with *pTERTmut* WHO grade II and III, in turn, received a diverse treatment regimen spanning wait-and-scan approaches to tumor resection with following radiochemotherapy. None of the different treatment approaches demonstrated a benefit in patient’s outcome when compared to other treatments. It remains to be noted that our sample size was limited. Prospective studies will need to address treatment approaches in such patients in the future.

In conclusion, our data show a similar extent of MGMT promotor methylation in patients with molecular GBM and patients with histopathological GBM. Methylation rates were similar in both cohorts defined by Sanger sequencing as well as MSP. MGMT methylation was associated with improved outcome in patients with histopathological GBM and showed a non-significant trend for improved outcome in molecular GBM patients treated with radio- or chemotherapy. However, in both cohorts higher numbers of methylated CpG sites were associated with a significant longer time to radiographic progression in case of first line treatment with alkylating chemotherapy. Randomized prospective studies of treatment algorithms accounting for MGMT promotor methylation status are urgently needed in patients with molecular GBM. Understanding the biological role of MGMT promotor status and its clinical impact in the presence of TERT promotor mutations and absence of IDH mutations in gliomas formerly assigned to WHO grade II and III may be of great importance for future therapeutic management of such patients.

## Data Availability

Data and material can be made available by request to the corresponding author.
